# Acute Pneumocystis jirovecii Pneumonia Due to Absolute Lymphopenia

**DOI:** 10.7759/cureus.22768

**Published:** 2022-03-02

**Authors:** Eric Hilker, Sachin M Patil, Rodger Wilhite, Zach Holliday

**Affiliations:** 1 Department of Internal Medicine, University of Missouri School of Medicine, Columbia, USA; 2 Infectious Disease, University of Missouri Health Care, Columbia, USA; 3 Pulmonary, Critical Care and Environmental Medicine, University of Missouri Health Care, Columbia, USA; 4 Department of Medicine, Division of Pulmonary and Critical Care, University of Missouri School of Medicine, Columbia, USA

**Keywords:** pneumocystis jiroveci pneumonia, hiv negative, prednisone, rheumatoid arthritis, lymphopenia

## Abstract

*Pneumocystis *pneumonia (PCP) is an opportunistic fungal infection associated with human immunodeficiency virus (HIV) infection as an acquired immunodeficiency syndrome (AIDS)-defining illness. The PCP incidence in patients with HIV has declined over the last few decades due to effective antiretroviral therapy and prophylaxis. The PCP incidence in HIV-negative patients has increased due to the increasing use of a wide array of immunosuppressants in cancer and autoimmune disease. PCP clinical course varies from patients with HIV in their clinical features, the severity of clinical presentation, and mortality. PCP in autoimmune diseases is rare, especially in rheumatoid arthritis (RA) in the United States of America (USA). Here, we describe an elderly Caucasian female with rheumatoid arthritis and left lung mucinous adenocarcinoma status post recent resection with no chemotherapy on a low dose of methotrexate (MTX) and prednisone presenting with acute hypoxic respiratory failure due to PCP from absolute lymphopenia.

## Introduction

*Pneumocystis jirovecii* is an opportunistic fungus responsible for acute *Pneumocystis* pneumonia (PCP) [1]. The PCP incidence in patients with HIV has reduced due to successful antiretroviral therapy and effective prophylaxis [2]. However, there has been a gradual increase in PCP cases in HIV-negative patients, especially autoimmune and/or inflammatory diseases (AIIDs) on immunosuppression [1]. The first case of PCP in rheumatoid arthritis (RA) was reported in 1983 and attributed to low-dose methotrexate (MTX) [3]. In patients with disease-modifying antirheumatic drug (DMARD)-naive RA, MTX is the initial choice for moderate to severe disease activity. An initial higher MTX dose is not associated with a better clinical outcome than a lower dose [4]. Low-dose MTX hinders disease progression and has a better side effect profile, making it a favorable therapeutic option [3]. An infrequent severe, life-threatening side effect of MTX is an increased risk of PCP. In non-HIV patients, the mortality rate of PCP is high (39.4%-59.1%) [5]. Here, we present an elderly Caucasian female with RA on immunomodulatory therapy manifesting with acute respiratory failure requiring mechanical ventilation due to PCP.

## Case presentation

A 68-year-old female with a past medical history significant for RA, right lung adenocarcinoma diagnosed in 2019 status post right upper and middle lobectomy with radical hilar mediastinal lymphadenectomy, prior smoker with 30 pack-years of smoking, and left lower lobe nodule status post wedge resection six weeks prior presented to an outside hospital emergency department for fever and dyspnea. Since the recent surgery, the patient admitted to generalized fatigue with poor oral intake, nausea, and vomiting. Home oral medications included Tylenol 1 g daily, MTX 12.5 mg weekly, omeprazole 20 mg daily, prednisone 5 mg daily, montelukast 10 mg daily, and ibuprofen 600 mg every eight hours as needed. Clinical examination was benign, and vital signs revealed tachycardia of 108 beats per minute (bpm), fever of 38.3°C, and oxygen saturation of 90% on room air. Laboratory results revealed leukocytosis and a negative rapid coronavirus disease 2019 (COVID-19) test and influenza antigen (Table 1).

**Table 1:** Laboratory results outside hospital emergency department. COVID-19: coronavirus disease 2019

Parameters	Results
White blood cell count (normal range: 4,500–11,000/mL)	15,500/mL (neutrophils: 84.4%, lymphocytes: 3.4%)
Absolute lymphocyte count (normal range: >2000 cells/mm^3^)	527 cells/mm^3^
Complete metabolic panel	Within normal limits
Lactic acid	1.6 mmol/L
COVID-19 rapid antigen test	Negative
Urine analysis with microscopy	Negative for urinary tract infection
Influenza antigen A and B	Negative
Urine pregnancy test	Negative
Blood cultures (two sets)	Negative
Urine culture	Negative

Chest X-ray two views revealed diffuse interstitial infiltrates bilaterally. Computed tomography (CT) of the chest with angiogram revealed left hilar and paraesophageal lymphadenopathy, scattered bilateral ground-glass opacities, a trace left pleural effusion, and no pulmonary embolism. The patient was treated with oxygen supplementation, antiemetics, and intravenous (IV) fluids and transferred to our institution for acute hypoxic respiratory failure.

On arrival, clinical examination revealed a fever (38.1°C), tachycardia (100 bpm), tachypnea (22/minute), and oxygen saturation of 93% on 2 liters (L) nasal cannula (NC) with no significant physical findings. She was started on ceftriaxone and azithromycin for community-acquired bacterial pneumonia. Laboratory results revealed leukocytosis, elevated lactic acid, negative respiratory pathogen panel, urine *Legionella*, and streptococcal antigen (Table 2).

**Table 2:** Laboratory results at our institution. COVID-19: coronavirus disease 2019, PCR: polymerase chain reaction

Parameters	Results
White blood cell count (normal range: 3,500–10,500/mL)	15,730/mL (day 1) (neutrophils: 81.2%, lymphocytes: 5%)
White blood cell count (normal range: 3,500–10,500/mL)	18,330/mL (day 3)
Absolute lymphocyte count (normal range: >2,000 cells/mm^3^)	1,620 cells/mm^3^ (day 1)
Creatinine (normal range: 0.5–1 mg/dL)	2 mg/dL (day 8)
Procalcitonin	1 ng/mL (day 6)
C-reactive protein (normal range: <0.4 mg/dL)	26.27 mg/dL (day 4)
Lactic acid (normal range: 0.5–2.2 mmol/L)	4.3 mmol/L (day 2)
Troponin T generation 5 (normal range: ≤14)	347 ng/L (day 3)
NT-pro brain natriuretic peptide (normal range: 0–125 pg/mL)	131 pg/mL (day 1)
Urine sodium	80 mmol/L (day 6)
Urine legionella and streptococcal antigen	Negative (day 2)
COVID-19 nasopharyngeal PCR	Negative (day 3)
Respiratory pathogen panel PCR	Negative (day 2)
Human immunodeficiency virus serology	Negative (day 3)

**Figure 1:**
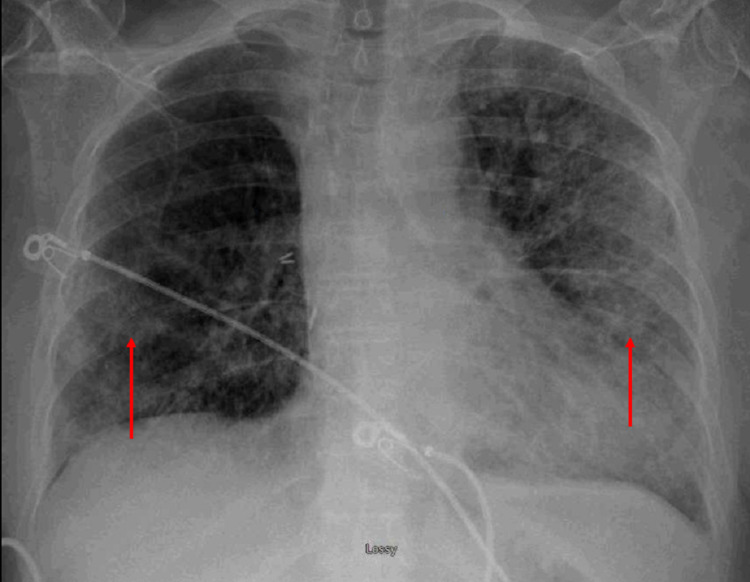
Portable chest X-ray revealed bilateral hazy airspace opacities (red arrows).

Portable chest X-ray revealed bilateral hazy airspace opacities (Figure 1).

**Figure 2:**
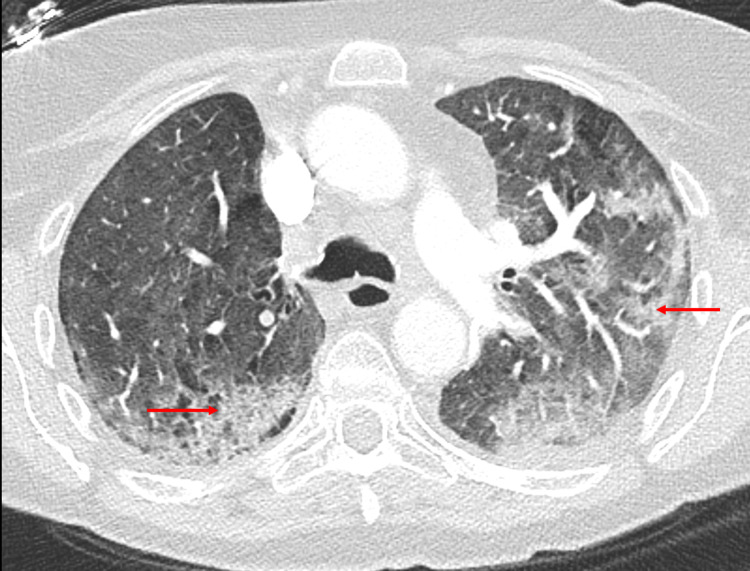
Chest CT pulmonary embolism protocol revealed new multifocal ground-glass interval consolidative opacities (red arrows), interlobular septal thickening, bilateral pleural effusions, and no pulmonary embolism.

**Figure 3:**
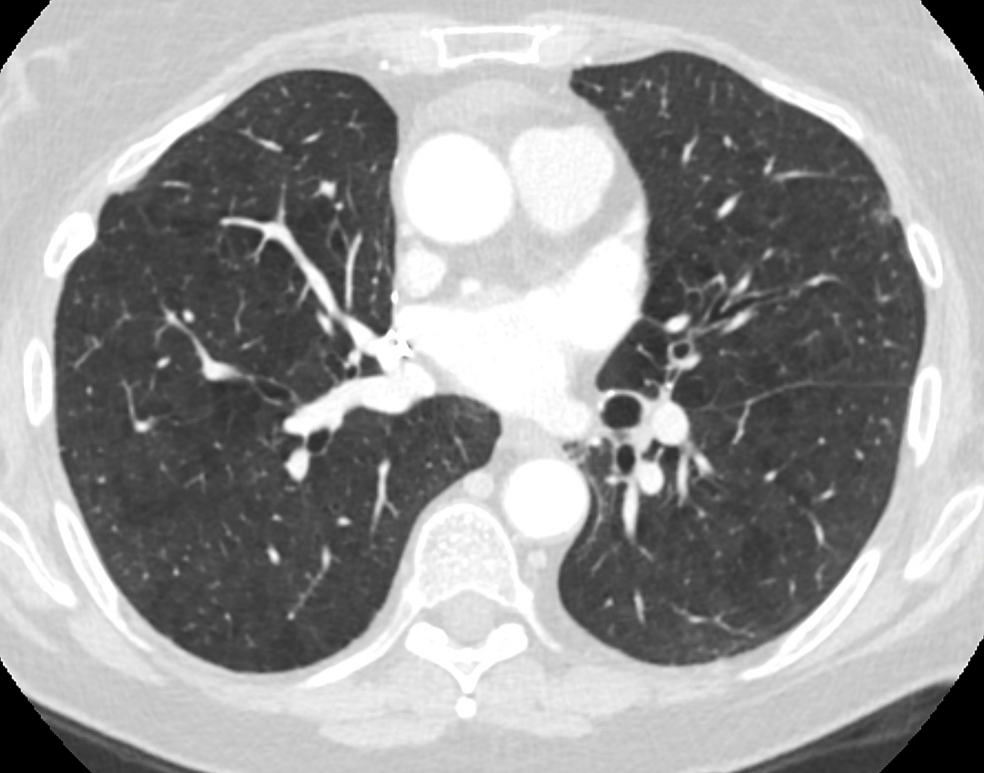
Chest CT with intravenous contrast done two months prior to the current admission revealed no airspace abnormalities.

Transthoracic echocardiogram revealed an ejection fraction of 40% with akinesis of mid-anterior, lateral, and inferior walls, hypokinesis of mid-inferoseptum, and basal distal segments suggestive of stress cardiomyopathy. She remained febrile over the next two days with worsening dyspnea and increased oxygen requirements (15 L/minute). Chest CT pulmonary embolism protocol revealed new multifocal ground-glass interval consolidative opacities, interlobular septal thickening, bilateral pleural effusions, and no pulmonary embolism, which was new compared to the chest CT done two months prior (Figures 2, 3).

On day 3, she was transferred to the medical intensive care unit for worsening hypoxia and tachypnea (38 beats per minute). She was placed intermittently on bilevel positive airway pressure (BIPAP)/high-flow nasal cannula (HFNC) and antibiotics broadened (vancomycin, Zosyn, and azithromycin). Blood work revealed leukocytosis with elevated inflammatory markers, troponin T, negative COVID-19 nasopharyngeal polymerase chain reaction test (PCR), and human immunodeficiency viral serology (HIV-1 antigen and HIV-1/2 antibody nonreactive). MTX-induced pneumonitis was suspected, and the patient was started on IV methylprednisolone 60 mg every six hours. The cardiology team recommended aspirin plus heparin drip for 48 hours for stress cardiomyopathy and non-ST-elevation myocardial infarction. Her respiratory effort increased, and she was intubated on day 6 with elevated serum creatinine and procalcitonin (Table 2). She then underwent bronchoscopy, which revealed bilaterally diffuse bronchiectatic airways, and 25 mL of lingula bronchoalveolar lavage (BAL) was obtained. Left lingula BAL cell count and pneumonia PCR panel were not suggestive of infection, and final cultures returned negative, except for the pending *Pneumocystis* workup (Table 3).

**Table 3:** Lingula bronchoalveolar fluid (BAL) results. PCR: polymerase chain reaction, GMS: Gömöri methenamine silver

Parameters	Results
Color and consistency	Pink and slightly cloudy
Cell count differential	Neutrophils: 1%, lymphocytes: 96%, and monocytes: 3%
White blood cell count	278/mcL
Red blood cell count	<3,000/mcL
Pneumonia PCR panel	Negative
Cultures (bacterial, fungal, and mycobacterial)	Negative
Pneumocystis PCR test	Negative
Cytology with GMS stain	Positive for *Pneumocystis* organisms

Chest X-ray on day 7 revealed left-sided pleural effusion, and by thoracentesis, 700 mL of straw-colored fluid was obtained. Left pleural fluid analysis revealed an exudative effusion based on Light’s criteria, and cultures returned negative (Table 4).

**Table 4:** Left pleural fluid analysis. LDH: lactate dehydrogenase

Parameters	Results
Color and consistency	Yellow and slightly cloudy
Neutrophils	1%
Lymphocytes	96%
Monocytes	3%
White blood cell count	2,304/mcL
Red blood cell count	3,000/mcL
pH	7.62
Glucose	192 mg/dL
LDH	423 units/L
Total protein	1.7 g/dL
Cultures (bacterial, fungal, and mycobacterial)	Negative
Flow cytometry CD4/CD8 ratio (≤3.50%)	4.68%
Light’s criteria	
Pleural fluid protein/serum protein	1.5 g/dL/5.5 g/dL = 0.309 (serum protein: 6.6–8.7 g/dL)
Pleural fluid LDH/serum LDH	423 units/L/723 units/L = 0.55 (serum LDH: 135–214 units/L)
Pleural fluid LDH > 2/3 serum LDH upper limit	2/3 × 214 = 142, (>142) 423

On the eighth day, the BAL Gömöri methenamine stain returned positive for *Pneumocystis*, whereas the PCR test was negative. Antibiotics were adjusted to IV trimethoprim/sulfamethoxazole (TMP-SMX), and corticosteroids were changed to prednisone on a tapering regimen recommended for PCP. The patient tolerated TMP-SMX with significant improvement in her oxygenation and was extubated on the 11th day to 8 L oxygen via NC. On the 13th day, the patient had mild hyperkalemia of 5.4 mmol/L with a creatinine of 1.8 mg/dL, for which TMP-SMX was changed to clindamycin and primaquine (normal glucose-6-phosphate dehydrogenase levels) by the infectious disease team. The patient was downgraded to the medical floor on 4 L oxygen via nasal cannula. She completed three weeks of antimicrobials and steroids with substantial improvement, followed by a discharge to an acute rehabilitation facility. She was transitioned to TMP-SMX single-strength (SS) one tablet daily for prophylaxis due to impending chemotherapy (left lower lobe nodule biopsy was positive for adenocarcinoma), and MTX was resumed at 7.5 mg weekly. She had normal serum creatinine and potassium levels at a follow-up visit six months later.

## Discussion

The PCP incidence in rheumatologic disorders is 1%-2% [6]. The PCP occurrence in the United States of America (USA) per 10,000 hospitalizations/year in RA is approximately two patients. The annual incidence rate of PCP in RA is 0.1%-0.3% in the USA, relatively infrequent compared to Japan [7,8]. PCP is the commonest opportunistic infection in patients with RA treated with low-dose MTX [9]. PCP risk factors include lymphopenia, corticosteroids, AIIDs, other immunosuppressive agents including biologics, age ≥ 65 years, solid tumors, and underlying lung disease [1,10]. For solid cancers, primary or metastatic lung cancer is associated with a higher risk for PCP [1]. Corticosteroids alone do not increase the PCP risk as a similar effect is not seen in all AIIDs, indicative of AIIDs being an independent risk factor [2]. Corticosteroid risk depends on the total dose, duration, and daily doses [5]. A daily corticosteroid dose of ≥20 mg prednisone equivalent for ≥4 weeks is considered a PCP risk factor [11]. Compared to other immunosuppressants, a daily dose of ≥10 mg prednisolone equivalent had the highest risk for PCP, while MTX risk was low [5]. In patients with RA treated with a daily corticosteroid dose of <10 mg prednisolone equivalent, concurrent MTX was a substantial causative factor for PCP occurrence [3,10]. PCP cumulative risk increases when corticosteroids are used simultaneously with other immunosuppressive medications [12]. Elderly patients with RA are at a high risk of asymptomatic colonization, which is frequent in patients on immunosuppression, cancer, and lung disease in healthy patients [9,13]. Colonization risk increases with a corticosteroid dose of >20 mg/day prednisolone equivalent due to significant pulmonary surfactant composition changes [11,13].

Although lymphopenia is a significant risk factor, the exact absolute lymphocyte count (ALC) cutoff value predisposing to PCP is unknown. Based on HIV studies, an ALC of <1000 cells/mm^3^ corresponds to CD4 count of <200 cells/mm^3^, whereas ALC of >2000 cells/mm^3 ^corresponds to CD4 count of >200 cells/mm^3^ [14]. Lymphocyte counts associated with a higher risk of developing PCP in patients with AIIDs include CD4 count of <250 cells/mm^3^, CD3+ count of <625 cells/mm^3^, pre-therapeutic ALC of <800 cells/mm^3^, and a three-month post-therapeutic ALC of <600 cells/mm^3^ [8,15]. MTX-related PCP is seen frequently in elderly patients [9]. Certain conditions enhance MTX-induced immunosuppression, including chronic kidney disease and simultaneous administration of NSAIDs, corticosteroids, or other immunosuppressants [16]. NSAIDs potentiate MTX toxicity by decreasing renal clearance and plasma protein binding and inhibiting hepatic metabolism [17]. At-risk patients acquire PCP either via the reactivation of latent infection or through environmental sources. Currently, no guidelines exist to guide chemoprophylaxis in patients with AIIDs. A meta-analysis suggested a 3.5% PCP incidence in any AIID condition for chemoprophylaxis. The number needed to treat for preventing a PCP in patients with RA is 1099, which is 30 times the number needed to harm [18]. Current expert opinion suggests primary chemoprophylaxis in patients with AIIDs treated with a prednisone equivalent of ≥20 mg/day for ≥1 month, and RA is a low-risk disease. A points-based system incorporates risk factors and suggests primary chemoprophylaxis if the patient’s total score is ≥5 points [2]. Due to the lower PCP incidence in patients with RA, an alternative is to monitor any of the following: CD4 counts directly or indirectly, CD3+ counts, pre-therapeutic ALC, and three-month post-therapeutic ALC to assess PCP risk in susceptible patients (male, elderly, and on dual immunosuppression). The recommended agent for chemoprophylaxis is TMP-SMX SS daily or two tablets of TMP-SMX SS three times a week [2]. Alternatives such as atovaquone or dapsone should be used in patients with allergies, liver abnormalities, or blood dyscrasias. If TMP-SMX is used as chemoprophylaxis in a patient with RA on MTX, then MTX dose adjustment plus complete blood cell count with differential and a complete metabolic panel should be used to detect adverse event risk.

Our patient had all the risk factors mentioned above with a total score of six [2]. Our patient was on MTX, prednisone, and NSAIDs simultaneously. Her ALC at presentation was 527 cells/mm^3^, indicating a CD4 count of <200 cells/mm^3^. Her clinical course was severe due to a delay in diagnosis, necessitating mechanical ventilation. Due to a lower incidence, PCP is less commonly suspected and considered in the differential diagnosis. Physicians should initiate prophylaxis if they have high-risk factors with no symptoms and consider PCP in patients with AIIDs on immunomodulatory therapy with respiratory complaints to evade the high mortality.

## Conclusions

With an increasing number of patients on MTX for AIIDs, PCP is becoming more prevalent with higher mortality rates. This case report highlights the importance of high clinical suspicion for PCP in the setting of low-dose MTX, especially in a patient with RA with high-risk factors. Although not recommended, periodical monitoring of AIID therapy in high-risk patients with lymphocyte counts or using the points-based scoring system is feasible. Although PCP is a rare side effect of low-dose MTX, the severity of clinical manifestation and high mortality warrant an increased understanding of risk factors to determine which patients require prophylactic therapy. An earlier diagnosis and treatment can improve the prognosis in these patients. Clinicians involved in the care of these patients should be prudent in considering the diagnosis of PCP as a differential when these patients infrequently manifest with respiratory complaints.
